# Impact of Comorbidities on Treatment Outcomes Among Saudi Patients With Colorectal Cancer: A Retrospective Study

**DOI:** 10.7759/cureus.100031

**Published:** 2025-12-24

**Authors:** Abdulla E Alshamari, Mohannad Y Aljarallah, Mohammed S Alotaibi, Abdulwahab A Alhussain, Sami A Alrasheedi, Sultan K Alruwaili, Nourah E Alsaiari, Ahmed A Mubarak, Samah A Aljohani, Nuwayyir Aldawsari

**Affiliations:** 1 General Surgery, King Salman Specialist Hospital, Hail, SAU

**Keywords:** charlson comorbidity index, colorectal cancer, comorbidities, diabetes mellitus, disease-free survival, hypertension, overall survival, retrospective cohort study, saudi arabia, treatment outcomes

## Abstract

Background: Colorectal cancer is a leading cause of cancer-related morbidity and mortality worldwide and is among the most common malignancies in Saudi Arabia. The high prevalence of chronic noncommunicable diseases in the Saudi population raises concern regarding the impact of comorbidities on colorectal cancer outcomes. However, local data evaluating the prognostic significance of comorbidity burden remain limited.

Methods: We conducted a retrospective cohort study across government health cluster hospitals in Saudi Arabia. Adult patients with histologically confirmed colorectal cancer diagnosed between January 2015 and December 2022 were included. Demographic, clinical, tumor-related, and treatment data were extracted from electronic medical records. Comorbidity burden was assessed using the Charlson Comorbidity Index (CCI). Patients were stratified by comorbidity status and CCI score (<3 vs. ≥3). The primary outcome was overall survival; secondary outcomes included disease-free survival, cancer-related mortality, postoperative complications, and treatment completion. Survival analyses were performed using Kaplan-Meier methods and Cox proportional-hazards models.

Results: A total of 312 patients were included (mean age: 61.8±11.9 years; 59.9% male). At least one comorbidity was present in 214 patients (68.6%), and 74 patients (23.7%) had a CCI score of 3 or higher. Hypertension (47.1%) and diabetes mellitus (42.3%) were the most common comorbidities. Advanced disease (stage III-IV) was observed in 215 patients (68.9%). Patients with higher comorbidity burden were less likely to undergo curative-intent surgery (60.8% vs. 83.2%) or complete adjuvant chemotherapy (35.1% vs. 62.6%) and experienced higher rates of postoperative complications (37.8% vs. 16.4%). Five-year overall survival was significantly lower among patients with comorbidities than those without comorbidities (48.2% vs. 66.7%, p=0.002), and disease-free survival was similarly reduced (42.6% vs. 59.1%, p=0.01).

Conclusions: Comorbidities are highly prevalent among Saudi patients with colorectal cancer and are associated with reduced treatment delivery and significantly poorer survival outcomes. Systematic assessment and proactive management of comorbidity burden should be integrated into colorectal cancer care to improve prognosis and optimize treatment outcomes.

## Introduction

Colorectal cancer (CRC) is one of the most commonly diagnosed malignancies worldwide and represents a major cause of cancer-related morbidity and mortality. CRC ranks among the leading cancers in both men and women, with a steadily increasing incidence over recent decades [1,2]. This rise has been attributed to population aging, lifestyle changes, and the growing prevalence of metabolic disorders, including obesity and diabetes mellitus (DM). Despite advances in screening, diagnosis, and treatment, outcomes for CRC remain heterogeneous, suggesting that factors beyond tumor stage and biology play an important role in determining prognosis [1-3].

Comorbid conditions are increasingly recognized as critical determinants of cancer outcomes, particularly in older populations. Chronic diseases, such as DM, cardiovascular disease (CVD), chronic kidney disease (CKD), and chronic respiratory disorders, may adversely affect survival through direct biological mechanisms or by limiting the delivery and tolerability of cancer-directed therapies [3-8]. Patients with significant comorbidity burden often experience higher rates of treatment-related complications, dose reductions, and early treatment discontinuation, which may compromise oncologic outcomes. Consequently, comorbidity assessment has become an essential component of comprehensive cancer care [2,7].

In the Saudi context, the burden of chronic noncommunicable diseases (NCDs) is among the highest globally, with high prevalence rates of DM, hypertension (HTN), and obesity. These conditions frequently coexist in patients with CRC and may influence both disease presentation and management [9-11]. However, data evaluating the impact of comorbidities on CRC prognosis in Saudi populations remains limited. Most available evidence is derived from Western cohorts, which may not fully reflect regional differences in disease epidemiology, healthcare delivery, and patient characteristics.

Understanding the prognostic significance of comorbidities in Saudi patients with CRC is essential for improving risk stratification and optimizing treatment strategies. By examining the relationship between comorbidity burden, treatment patterns, and survival outcomes within a real-world health cluster setting, this study aimed to address an important gap in the local literature and to inform more individualized and multidisciplinary approaches to CRC care in Saudi Arabia.

## Materials and methods

Study design and setting

This retrospective cohort study was conducted within hospitals of a governmental health cluster in Saudi Arabia. The health cluster comprises tertiary and secondary care institutions that provide oncologic services, including diagnosis, surgical management, chemotherapy, and long-term follow-up for patients with colorectal cancer (CRC). The study period included patients diagnosed and treated between January 2015 and December 2022.

Study population

Adult patients (≥18 years of age) with a histologically confirmed diagnosis of CRC were eligible for inclusion. Patients were identified through hospital cancer registries and electronic medical records (EMRs). Individuals were included if they received an initial diagnostic evaluation and follow-up care within the health cluster hospitals. Patients with incomplete medical records, unknown tumor stage at diagnosis, or missing key outcome data were excluded from the analysis.

Data collection and variables

Clinical, demographic, and oncologic data were extracted retrospectively from EMRs using a standardized data collection form. Collected demographic variables included age at diagnosis, sex, body mass index (BMI), smoking status, family history of CRC, and Eastern Cooperative Oncology Group (ECOG) performance status. Comorbidities were recorded based on documented physician diagnoses and included diabetes mellitus (DM), HTN, dyslipidemia, ischemic heart disease (IHD), chronic kidney disease (CKD), chronic respiratory disease, chronic liver disease (CLD), and other relevant medical conditions. The comorbidity burden was quantified using the Charlson Comorbidity Index (CCI), which was calculated for each patient at the time of cancer diagnosis.

Tumor-related variables included tumor location, histologic subtype and grade, tumor-node-metastasis (TNM) stage at diagnosis, presence of lymphovascular or perineural invasion, and metastatic disease at presentation. Treatment-related variables comprised type and intent of surgery, use of chemotherapy and radiotherapy (RT), treatment completion status, dose modifications, postoperative complications, and need for intensive care unit (ICU) admission.

Outcome measures

The primary outcome was overall survival (OS), defined as the time from CRC diagnosis to death from any cause or last follow-up. Secondary outcomes included disease-free survival (DFS), defined as the time from curative-intent treatment to documented recurrence or death, cancer-related mortality, postoperative complications, and treatment tolerance, including chemotherapy completion and dose reduction. Follow-up duration was calculated as the interval from diagnosis to the date of the last documented clinical encounter.

Statistical analysis

Continuous variables were summarized as means with standard deviations (SDs) or medians with interquartile ranges (IQRs), as appropriate, and categorical variables were expressed as frequencies and percentages. Patients were stratified according to comorbidity burden using the CCI (CCI <3 vs. CCI ≥3) and by the presence or absence of comorbidities. Comparisons between groups were performed using the Student’s t-test or Mann-Whitney U test for continuous variables and the chi-square test or Fisher’s exact test for categorical variables.

Survival outcomes were estimated using the Kaplan-Meier method and compared between groups with the log-rank test. Cox proportional hazards regression models were used to assess the association between comorbidity burden and survival outcomes, adjusting for clinically relevant covariates, including age, sex, tumor stage, and treatment modality. Hazard ratios (HRs) with 95% confidence intervals (CIs) were reported. A two-sided p-value of less than 0.05 was considered statistically significant. Statistical analyses were performed using SPSS software version 26.0 (Armonk, NY: IBM Corp.).

## Results

Study population and baseline characteristics

A total of 312 patients with CRC were included in the study. The mean age at diagnosis was 61.8±11.9 years, and 134 patients (42.9%) were aged 65 years or older. Men accounted for 187 patients (59.9%) of the study population. The mean BMI was 28.7±5.4 kg/m², with obesity (BMI ≥30) present in 112 patients (35.9%). Seventy-eight patients (25.0%) were current or former smokers, and 69 patients (22.1%) had an Eastern Cooperative Oncology Group (ECOG) performance status of 2 or higher. A family history of CRC was reported in 41 patients (13.1%) (Table 1).

**Table 1:** Baseline characteristics of the study population. Data are presented for 312 patients with colorectal cancer (CRC) treated in hospitals of a Saudi Arabian health cluster. Values are expressed as mean±standard deviation (SD) for continuous variables and number (%) for categorical variables. Body mass index (BMI) was calculated as weight in kilograms divided by height in meters squared. Eastern Cooperative Oncology Group (ECOG) performance status ≥2 indicates patients with limited functional status.

Characteristics	Total (n=312)
Age, mean±SD (years)	61.8±11.9
Age ≥65 years, n (%)	134 (42.9)
Male sex, n (%)	187 (59.9)
BMI, mean±SD (kg/m²)	28.7±5.4
Obesity (BMI ≥30), n (%)	112 (35.9)
Current or former smoker, n (%)	78 (25.0)
ECOG performance status ≥2, n (%)	69 (22.1)
Family history of colorectal cancer, n (%)	41 (13.1)

Comorbidity profile

Overall, 214 patients (68.6%) had at least one comorbid condition. HTN was the most common comorbidity, affecting 147 patients (47.1%), followed by DM in 132 patients (42.3%) and dyslipidemia in 101 patients (32.4%). Ischemic heart disease (IHD) was present in 46 patients (14.7%), CKD stage 3 or higher in 28 patients (9.0%), chronic respiratory disease in 33 patients (10.6%), and CLD in 21 patients (6.7%). Multiple comorbidities were frequent, with 96 patients (30.8%) having two or more comorbid conditions, and 74 patients (23.7%) having a CCI score of 3 or higher (Table 2).

**Table 2:** Prevalence of comorbidities. This table shows the frequency of comorbid conditions among 312 patients with colorectal cancer (CRC). Comorbidity burden was quantified using the Charlson Comorbidity Index (CCI), with a score ≥3 representing high comorbidity burden. Chronic kidney disease (CKD) was defined as stage 3 or higher. Chronic obstructive pulmonary disease (COPD) includes asthma. Values are presented as a number (%).

Comorbidities	n (%)
Any comorbidity	214 (68.6)
Diabetes mellitus	132 (42.3)
Hypertension	147 (47.1)
Dyslipidemia	101 (32.4)
Ischemic heart disease	46 (14.7)
Chronic kidney disease (stage ≥3)	28 (9.0)
Chronic liver disease	21 (6.7)
COPD or asthma	33 (10.6)
≥2 comorbidities	96 (30.8)
Charlson Comorbidity Index ≥3	74 (23.7)

Tumor characteristics at diagnosis

Primary tumors were located in the right colon in 96 patients (30.8%), the left colon in 124 patients (39.7%), and the rectum in 92 patients (29.5%). Histologically, 62 tumors (19.9%) were well differentiated, 191 (61.2%) were moderately differentiated, and 59 (18.9%) were poorly differentiated. At presentation, 97 patients (31.1%) had stage I-II disease, 121 (38.8%) had stage III disease, and 94 (30.1%) had stage IV disease. Metastatic disease at diagnosis was identified in 94 patients (30.1%) (Table 3).

**Table 3:** Tumor characteristics at diagnosis. Tumor location, histologic grade, stage at diagnosis, and presence of metastatic disease are shown for 312 patients with CRC. Histologic grade is classified as well, moderately, or poorly differentiated. Stage at diagnosis is based on the tumor-node-metastasis (TNM) classification system. Metastatic disease at presentation indicates evidence of distant metastasis at the time of diagnosis. Values are presented as number (%).

Variables		n (%)
Tumor location	Right colon	96 (30.8)
	Left colon	124 (39.7)
	Rectum	92 (29.5)
Histologic grade	Well differentiated	62 (19.9)
	Moderately differentiated	191 (61.2)
	Poorly differentiated	59 (18.9)
Stage at diagnosis	I-II	97 (31.1)
	III	121 (38.8)
	IV	94 (30.1)
	Metastatic disease at presentation	94 (30.1)

Treatment according to the comorbidity burden

Among patients with a CCI score below 3 (n=238), curative-intent surgery was performed in 198 patients (83.2%), compared with 45 of 74 patients (60.8%) with a CCI score of 3 or higher. Adjuvant chemotherapy was administered to 164 patients (68.9%) in the lower-CCI group and to 33 patients (44.6%) in the higher-CCI group. Completion of planned chemotherapy occurred in 149 patients (62.6%) with CCI <3 vs. 26 patients (35.1%) with CCI ≥3. Dose reductions were more common in patients with higher comorbidity burden (29 patients {39.2%} vs. 41 patients {17.2%}), as were postoperative complications (28 patients {37.8%} vs. 39 patients {16.4%}) (Table 4).

**Table 4:** Treatment characteristics according to comorbidity burden. This table presents the treatment modalities and outcomes stratified by Charlson Comorbidity Index (CCI) score (<3 vs. ≥3) among 312 patients with CRC. Curative surgery indicates surgery performed with curative intent. Adjuvant chemotherapy refers to systemic therapy administered after surgery. Chemotherapy completion represents patients who received the planned full regimen. Dose reduction required indicates any chemotherapy dose reduction due to toxicity or comorbidities. Postoperative complications include any major complication within 30 days of surgery. Values are presented as number (%).

Treatment variables	CCI <3 (n=238)	CCI ≥3 (n=74)
Curative surgery, n (%)	198 (83.2)	45 (60.8)
Adjuvant chemotherapy, n (%)	164 (68.9)	33 (44.6)
Chemotherapy completion, n (%)	149 (62.6)	26 (35.1)
Dose reduction required, n (%)	41 (17.2)	29 (39.2)
Postoperative complications, n (%)	39 (16.4)	28 (37.8)

Survival outcomes

After a median follow-up of 38 months in patients without comorbidities and 34 months in those with at least one comorbidity, survival outcomes differed significantly between groups. The three-year overall survival (OS) rate was 78.4% among patients without comorbidities compared with 61.9% among those with one or more comorbid conditions. Five-year OS was 66.7% in patients without comorbidities and 48.2% in patients with comorbidities (p=0.002). Disease-free survival (DFS) was also higher in patients without comorbidities (59.1% vs. 42.6%, p=0.01). Cancer-related mortality occurred in 29 patients (29.6%) without comorbidities and in 92 patients (43.0%) with comorbidities (p=0.03) (Table 5).

**Table 5:** Survival outcomes according to comorbidity status. Overall survival (OS) and disease-free survival (DFS) are presented according to presence or absence of comorbidities in 312 patients with CRC. OS is defined as time from CRC diagnosis to death from any cause or last follow-up. DFS is defined as time from curative-intent treatment to recurrence or death. Cancer-related mortality refers to deaths attributed to CRC. Median follow-up is reported in months. Values are presented as number (%) or median. P-values indicate significance of differences between groups.

Outcomes	No comorbidity (n=98)	≥1 comorbidity (n=214)	p-Value
Median follow-up, months	38	34	-
3-year overall survival (%)	78.4	61.9	0.004
5-year overall survival (%)	66.7	48.2	0.002
Disease-free survival (%)	59.1	42.6	0.01
Cancer-related mortality, n (%)	29 (29.6)	92 (43.0)	0.03

## Discussion

In this retrospective cohort study conducted within Saudi Arabian health cluster hospitals, comorbidities were highly prevalent among patients with CRC and were associated with adverse clinical outcomes. Nearly two-thirds of patients had at least one comorbid condition, and approximately one-quarter had a high comorbidity burden as reflected by a CCI score of 3 or higher. Patients with comorbidities experienced poorer OS and DFS, higher cancer-related mortality, and reduced likelihood of completing planned oncologic treatment, underscoring the clinically significant impact of comorbidity burden on prognosis.

The prevalence of DM and HTN observed in this cohort reflects the high burden of metabolic and cardiovascular disease (CVD) in the Saudi population and is consistent with national epidemiologic data. These conditions may influence CRC outcomes through multiple mechanisms, including chronic inflammation, impaired immune function, and reduced physiologic reserve. In addition, comorbidities may limit the intensity of cancer-directed therapies that patients can safely receive, thereby indirectly affecting survival.

Advanced-stage disease was common at presentation, with nearly 70% of patients diagnosed at stage III or IV. This finding aligns with regional reports indicating delayed diagnosis of CRC in Saudi Arabia, likely related to limited screening uptake and delayed symptom recognition. The coexistence of multiple chronic diseases may further contribute to diagnostic delays by masking cancer-related symptoms or prioritizing management of nonmalignant conditions [10-13].

Treatment disparities according to comorbidity burden were a key finding of this study. Patients with higher CCI scores were significantly less likely to undergo curative-intent surgery or receive adjuvant chemotherapy and were substantially more likely to experience postoperative complications and require chemotherapy dose reductions. These observations suggest that comorbidities influence both clinician decision-making and treatment tolerance. Importantly, reduced treatment completion among patients with high comorbidity burden likely contributed to the observed survival differences, highlighting comorbidities as both direct and indirect prognostic factors [5-9].

Survival analysis demonstrated a clear and clinically meaningful decrement in outcomes among patients with comorbidities. Five-year OS was nearly 20 percentage points lower among patients with at least one comorbid condition compared with those without comorbidities. DFS showed a similar pattern, and cancer-related mortality was significantly higher in patients with comorbidities. These findings are consistent with international literature demonstrating that comorbidity burden independently predicts poorer survival in CRC, even after adjustment for age and tumor stage [1-7].

The results of this study have important clinical implications. Early identification and optimal management of comorbid conditions may improve treatment tolerance and outcomes in patients with CRC. Multidisciplinary care models that integrate oncology, internal medicine, and subspecialty services may be particularly beneficial for patients with high comorbidity burden. Furthermore, incorporating standardized comorbidity assessment tools, such as the CCI, into routine oncologic evaluation may aid in risk stratification and individualized treatment planning.

Several limitations should be acknowledged. The retrospective design introduces the potential for selection bias and incomplete documentation. Residual confounding by unmeasured variables, such as socioeconomic status or frailty, cannot be excluded. In addition, data were derived from a single health cluster, which may limit generalizability to other healthcare settings. Nevertheless, the multicenter nature of the health cluster and the relatively large sample size strengthen the validity of the findings.

## Conclusions

In this retrospective study of CRC patients treated within Saudi Arabian health cluster hospitals, comorbidities were common and were associated with significantly poorer clinical outcomes. A higher comorbidity burden was linked to reduced likelihood of receiving and completing curative-intent treatment, increased postoperative complications, and lower OS and DFS. These findings highlight the importance of systematic assessment and proactive management of comorbid conditions as an integral component of CRC care. Incorporating comorbidity-based risk stratification into clinical decision-making and adopting multidisciplinary care approaches may help optimize treatment delivery and improve prognosis for CRC patients in Saudi Arabia.

## References

[1] Boakye D, Walter V, Jansen L, Martens UM, Chang-Claude J, Hoffmeister M, Brenner H (2020). Magnitude of the age-advancement effect of comorbidities in colorectal cancer prognosis. J Natl Compr Canc Netw.

[2] Boakye D, Jansen L, Schneider M, Chang-Claude J, Hoffmeister M, Brenner H (2019). Personalizing the prediction of colorectal cancer prognosis by incorporating comorbidities and functional status into prognostic nomograms. Cancers (Basel).

[3] Morishima T, Matsumoto Y, Koeda N (2019). Impact of comorbidities on survival in gastric, colorectal, and lung cancer patients. J Epidemiol.

[4] Boakye D, Rillmann B, Walter V, Jansen L, Hoffmeister M, Brenner H (2018). Impact of comorbidity and frailty on prognosis in colorectal cancer patients: a systematic review and meta-analysis. Cancer Treat Rev.

[5] Voutsadakis IA (2017). Obesity and diabetes as prognostic factors in patients with colorectal cancer. Diabetes Metab Syndr.

[6] Sun G, Li Y, Peng Y (2018). Can sarcopenia be a predictor of prognosis for patients with non-metastatic colorectal cancer? A systematic review and meta-analysis. Int J Colorectal Dis.

[7] Fataftah J, El-Hammuri N, Gharaibeh M (2024). Association of patient comorbidities with colorectal cancer site as detected by computed tomography scan and colonoscopy: a retrospective study. Medicine (Baltimore).

[8] Numata M, Sawazaki S, Morita J (2018). Comparison of laparoscopic and open surgery for colorectal cancer in patients with severe comorbidities. Anticancer Res.

[9] Tammana VS, Laiyemo AO (2014). Colorectal cancer disparities: issues, controversies and solutions. World J Gastroenterol.

[10] Hodgson DC, Fuchs CS, Ayanian JZ (2001). Impact of patient and provider characteristics on the treatment and outcomes of colorectal cancer. J Natl Cancer Inst.

[11] Faivre J, Lemmens VE, Quipourt V, Bouvier AM (2007). Management and survival of colorectal cancer in the elderly in population-based studies. Eur J Cancer.

[12] Deng L, Gui Z, Zhao L, Wang J, Shen L (2012). Diabetes mellitus and the incidence of colorectal cancer: an updated systematic review and meta-analysis. Dig Dis Sci.

[13] Lagra F, Karastergiou K, Delithanasis I, Koutsika E, Katsikas I, Papadopoulou-Zekeridou P (2004). Obesity and colorectal cancer. Tech Coloproctol.

